# Effect of in situ VSi_2_ and SiC phases on the sintering behavior and the mechanical properties of HfB_2_-based composites

**DOI:** 10.1038/s41598-020-73295-7

**Published:** 2020-10-06

**Authors:** Soheil Ghadami, Ehsan Taheri-Nassaj, Hamid Reza Baharvandi, Farzin Ghadami

**Affiliations:** 1 Department of Materials Science and Engineering, Tarbiat Modares University, PO Box 14115-143, Tehran, Iran; 2 School of Metallurgy and Materials, College of Engineering, University of Tehran, Tehran, Iran

**Keywords:** Engineering, Materials science, Chemical physics

## Abstract

In situ HfB_2_–SiC–VSi_2_ composite was fabricated by reactive pressureless sintering at the temperature of 2150 °C for 4 h under a vacuum atmosphere. In situ SiC and VSi_2_ reinforcements were formed using VC and Si powders as starting materials according to the following reaction: VC + 3Si = SiC + VSi_2_. Microstructural studies and thermodynamic calculations revealed that in situ VSi_2_ and SiC phases were mostly formed and homogeneously distributed in HfB_2_ skeleton. The results showed that the density of in situ HfB_2_–SiC–VSi_2_ composite was 98%. Besides, the mechanical properties of the composite were effectively enhanced by the formation of in situ second phases. The Vickers hardness and the fracture toughness of the composite reached 20.1 GPa and 5.8 MPa m^−1/2^, respectively.

## Introduction

Advanced ceramics and protective coatings for high temperature applications have been recently attracted^1–11^. With a high melting point (about 3380 °C), high thermal and electrical conductivity, excellent strength at the severe environment, and brilliant thermal shock resistance, HfB_2_ is one of the ultra-high temperature ceramics (UHTCs). Due to its excellent properties, it has been considered for high-temperature applications such as nose cone and the leading edge of hypersonic flight vehicles and advanced rocket motors^12–14^. Recently, many studies have been undertaken to densify HfB_2_^15–17^. Because of the low self-diffusion coefficients and tightly covalent bonding, generally, pressure-assisted methods such as Spark plasma sintering (SPS), and Hot pressing (HP) are applied for consolidation of HfB_2_-based composites. However, using these methods restrict geometrical dimensions, especially for complex-shaped specimens. Reactive pressureless sintering is one of the practical methods to fabricate near-net-shape HfB_2_-based composites where matrix or reinforcement phases are in situ formed.

Brochu et al.^18^ densified ZrB_2_ ceramic by reactive pressureless sintering method using Zr and B powders as starting materials. However, they did not use any additive for the densification of ZrB_2_; the maximum density was reported about 79% for monolithic ZrB_2_.

Wang et al.^19^ reported the relative density of 97.2% for B_4_C–SiC–TiB_2_ composite fabricated by reactive pressureless sintering method.

Zhang et al.^20^ fabricated Ta_0.8_Hf_0.2_C–SiC composite using HfSi_2_, TaC, and carbon black powders by reactive pressureless sintering method at 2200 °C. The relative density of the composite was reported about 99%.

It has been reported that the oxide impurities (HfO_2_, B_2_O_3_) of HfB_2_ starting powder can prevent the densification of HfB_2_ ceramic^21^. Therefore, removing the oxide impurities and reaching full dense HfB_2_-based composites has been a challenging issue for researchers. Some additives or reinforcements have been suggested to enhance the sinterability and mechanical properties of UHTCs. In an attempt to increase the sintered density of UHTC-based composites, some researchers used oxide and non-oxide additives such as Y_2_O_3_^22^, Ta^23^, Al^24^, TaSi_2_^25^, and MoSi_2_^26^. Among them, SiC is an additive that has been commonly used due to its capability to improve the mechanical properties as well as the oxidation resistance of transition metal borides^27,28^. Moreover, silicides have been added to HfB_2_ to improve its mechanical properties owing to such superior properties as excellent creep resistance and oxidation behavior.

The addition of VSi_2_ for enhancing properties of UHTC is a novel idea. However, the sintering process of HfB_2_ ceramic with other silicides has been accomplished by other researchers. For example, Sciti et al.^25^ densified UHTC-based composites containing 3 vol% silicides of molybdenum or tantalum as sintering additives. They have reached the fracture toughness of 5.1 MPa m^1/2^ for the HfB_2_–TaSi_2_ composites as well as 4.4 MPa m^1/2^ for the HfB_2_–MoSi_2_ composites. In other research, Zhang et al.^29^ fabricated ZrB_2_–WSi_2_ composite via hot pressing method. They reported the fracture toughness of 3.5 MPa m^1/2^ for the composite. SiC and VSi_2_ could be suitable additives for HfB_2_-based composites, due to their low density, high thermal conductivity, and excellent oxidation and creep resistance^30,31^.

The aim of this work is the fabrication and properties evaluation of HfB_2_–SiC–VSi_2_ composite which is fabricated by HfB_2_, VC, and Si powders via reactive pressureless sintering method. We investigate the effect of in situ VSi_2_ and SiC phases on the densification, microstructure, and mechanical properties of HfB_2_–SiC–VSi_2_ composite.

## Experimental methods

In order to fabricate the HfB_2_–15 vol%SiC–15vol%VSi_2_ composite, the commercial HfB_2_, VC, and Si powders were used as starting materials. The characteristics of starting powders are listed in Table 1. Calculations of volume fractions were performed to define the composition of the composite. The powders were milled by a high-energy planetary mill for 5 h in ethanol medium. WC–Co cup and balls were selected and a speed ratio of the milling process was defined 300 rpm. The weight ratio of powders to balls was determined 1:3. For removing ethanol from mixed powders, the drying process was accomplished for 24 h in air. Cylindrical specimens (Φ25 × 8 mm^2^) without any binders were cold-pressed by uniaxial pressing at 50 MPa and then were cold isostatically pressed at 300 MPa. Reactive pressureless sintering process was performed in a commercial graphite resistance heating furnace at 2150 °C for 4 h under a vacuum atmosphere of 0.05 mbar. For completing the formation of in situ phases, a heating rate was decreased from 1150 to 1350 °C according to the reaction (3). Table 2 shows the main features and the sintering conditions for the sintered composite.

**Table 1:** Specification of the starting powders before sintering.

Row material	Purity (%)	Mean particle size (µm)	Impurities	Vendor
HfB_2_	95	20	HfO_2_	Beijing Cerametek Materials (China)
VC	99.9	2	–	Merck (Germany)
Si	99.5	10	–	Merck (Germany)

**Table 2:** Sintering conditions and properties of HfB_2_–SiC–VSi_2_ composite.

SiC (vol%)	VSi_2_ (vol%)	Temperature/Vacuum pressure/Dwell time of Reactive pressureless sintering process (°C/mbar/h)	Relative green density (%)	Relative density (%)	Elastic modulus (GPa)	Vickers Hardness (GPa)	Matrix Grain Size (µm)	Fracture Toughness (MPa m^1/2^)
10	20	2150/0.05/4	60	98	401.3 ± 1.3	20.1 ± 1.1	10	5.8 ± 0.33

Before the sintering process, the green density of the composite was measured by the ratio between the mass and the volume of the sintered specimens according to dimensional measurements. It should be noted that the relative green density was reported by the ratio between green and theoretical densities. The theoretical density of the composite was calculated based on the final composition after the sintering process (see Sect. Densification and microstructure and Fig. 2) by the rule of mixture according to theoretical densities of 11.2 g/cm^3^ for HfB_2_, 3.2 g/cm^3^ for SiC, 4.42 g/cm^3^ for VSi_2_, and 12.2 g/cm^3^ for HfC. The bulk density of sintered samples was measured using Archimedes method. Hence, the relative density of the sintered specimens was reported by the ratio between the bulk and theoretical densities. Young's modulus was determined through ultrasonic testing at 25 °C according to the ASTM C1198^32^ by sound velocity using the TC600 model thickness measuring apparatus. The Vickers hardness test was carried out on the polished surfaces of the sintered specimens by a Vickers indenter with 0.3 kg applied load for 10 s^33^:

1 $$Hv=1.854\frac{P}{{d}^{2}}$$

where Hv is the Vickers hardness (GPa), P refers to the applied force for indentation (N), and d means the average diagonal length of indent (m).

The fracture toughness of the sintered specimens was calculated using Evans and Charles’s equation^34^:

2 $$K{\text{IC}}=0.16 {(c/a)}^{-3/2}{ (Ha}^{1/2})$$

where $KIC$ refers to the fracture toughness (MPa m^−1/2^), H means Vickers hardness (GPa), *c* is the average half-length of the crack acquired in the tips of the Vickers marks (m), and *a* is the average half-length of indentation diagonal (m). The fracture toughness was evaluated by the applied load of 20 kg.

To the accuracy of the result, five specimens for HfB_2_–SiC–VSi_2_ composite were tested and ten measurements were repeated for each specimen. Moreover, the microstructural observation was examined on the mirror-like surfaces of sintered specimens by field emission scanning electron microscope (FESEM, TESCAN, Model: MIRA3) equipped with energy-dispersive spectroscopy (EDS). Besides, to ensure reliable results, the microstructural analysis was done on different parts of the specimens. The phase composition was determined by X-ray diffraction analysis (XRD, Philips, Model: X’Pert MPD, Tube: Co, and λ: 1.78897 Å). The grain size of the sintered composite was estimated by the image analysis (ImageJ software). To determine the possibility of in situ formations of phases during the sintering process, thermodynamic calculations were performed using HSC software. The final composition after sintering was calculated by ImageJ analyzing software. For this purpose, ten random images of SEM micrographs at different magnifications were selected and evaluated.

## Result and discussion

### Densification and microstructure

**Figure 1:**
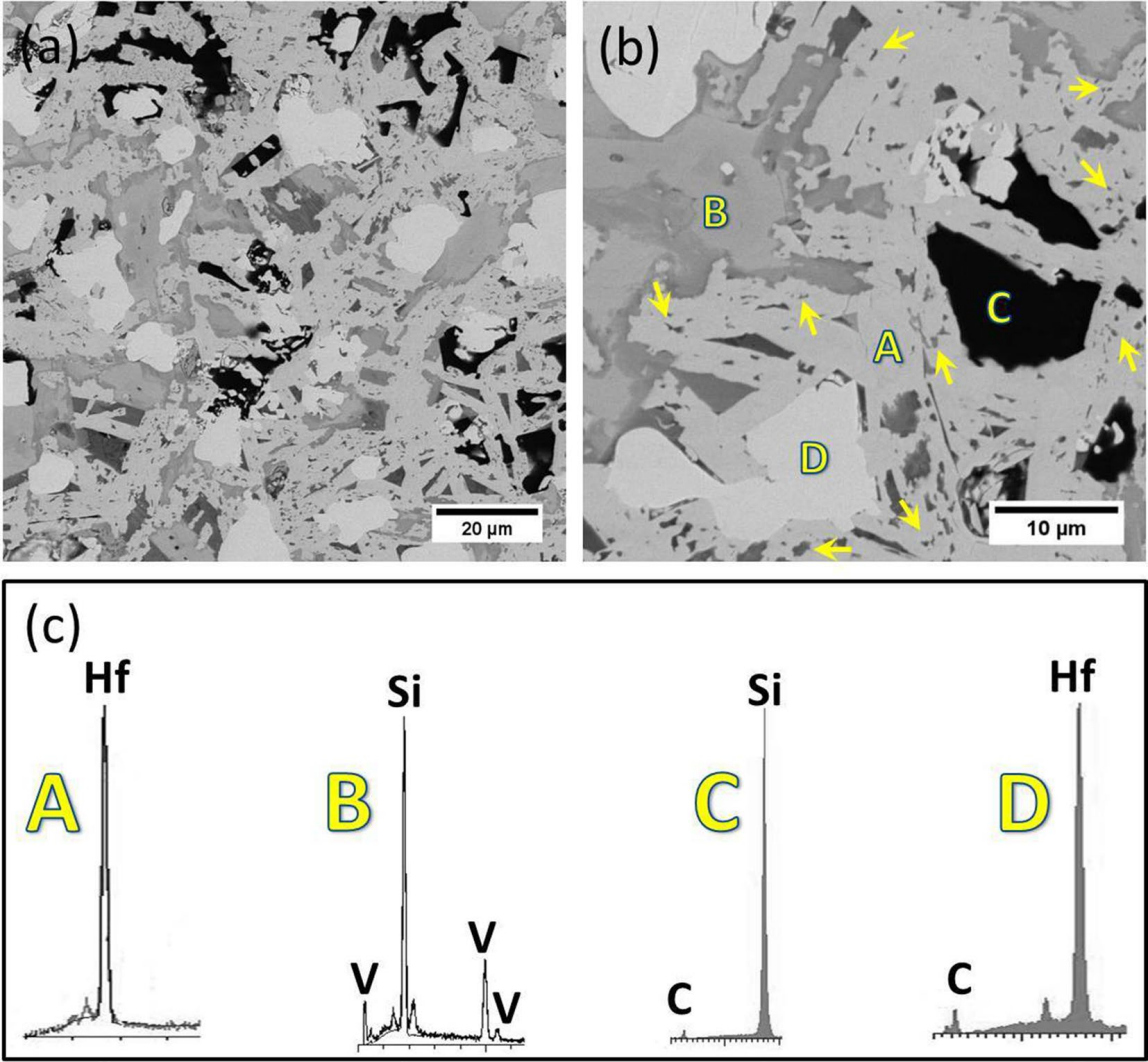
Field emission SEM micrographs of pressureless sintered HfB_2_–SiC–VSi_2_ composite at 2150 °C (**a**) low magnification, (**b**) high magnification, and (**c**) EDS patterns of spot A, spot B, spot C, and spot D. Ultra-fine grains indicated by arrows in (**b**) have chemical composition according to B.

The relative green and relative densities of reactive pressureless sintered HfB_2_–SiC–VSi_2_ composite are presented in Table 2. The relative density of the composite reached 98%. FESEM image of the microstructure of the pressureless sintered composite is shown in Fig. 1. It has been reported that diffusion rate and porosity mobility are enhanced by increasing the sintering temperature which finally causes the reduction of cavities in the sintered composite^35^. The very small amount of porosity is observed in the microstructure after the sintering process which confirms that the temperature of the sintering process (~ 2150 °C) was adequate to remove most of the porosities. On the other hand, the mobility of the grain boundary of HfB_2_ was decreased by in situ formations of SiC and VSi_2_ phases alongside HfB_2_ grains. The average HfB_2_ grain size was estimated about 10 µm. Some ultra-fine grains inside HfB_2_ phase could be found and EDS analysis revealed them to be VSi_2_. Due to the 1677 °C melting point of VSi_2_, it seems that the VSi_2_ was molten during the sintering process. The molten VSi_2_ flowed through the capillaries and filled the pores. Based on this scenario, these ultra-fine grains were recrystallized VSi_2_ which were located at pores and finally led to an improvement in the density of the composite. Aside from VSi_2_ phase, three regions are distinguished in the microstructure of the composite. Black regions are SiC, white regions are HfC, and the light regions are HfB_2_ according to EDS analysis. This result is in excellent agreement with the previous study of the fabrication of in situ HfB_2_-based composites^21^.

**Figure 2:**
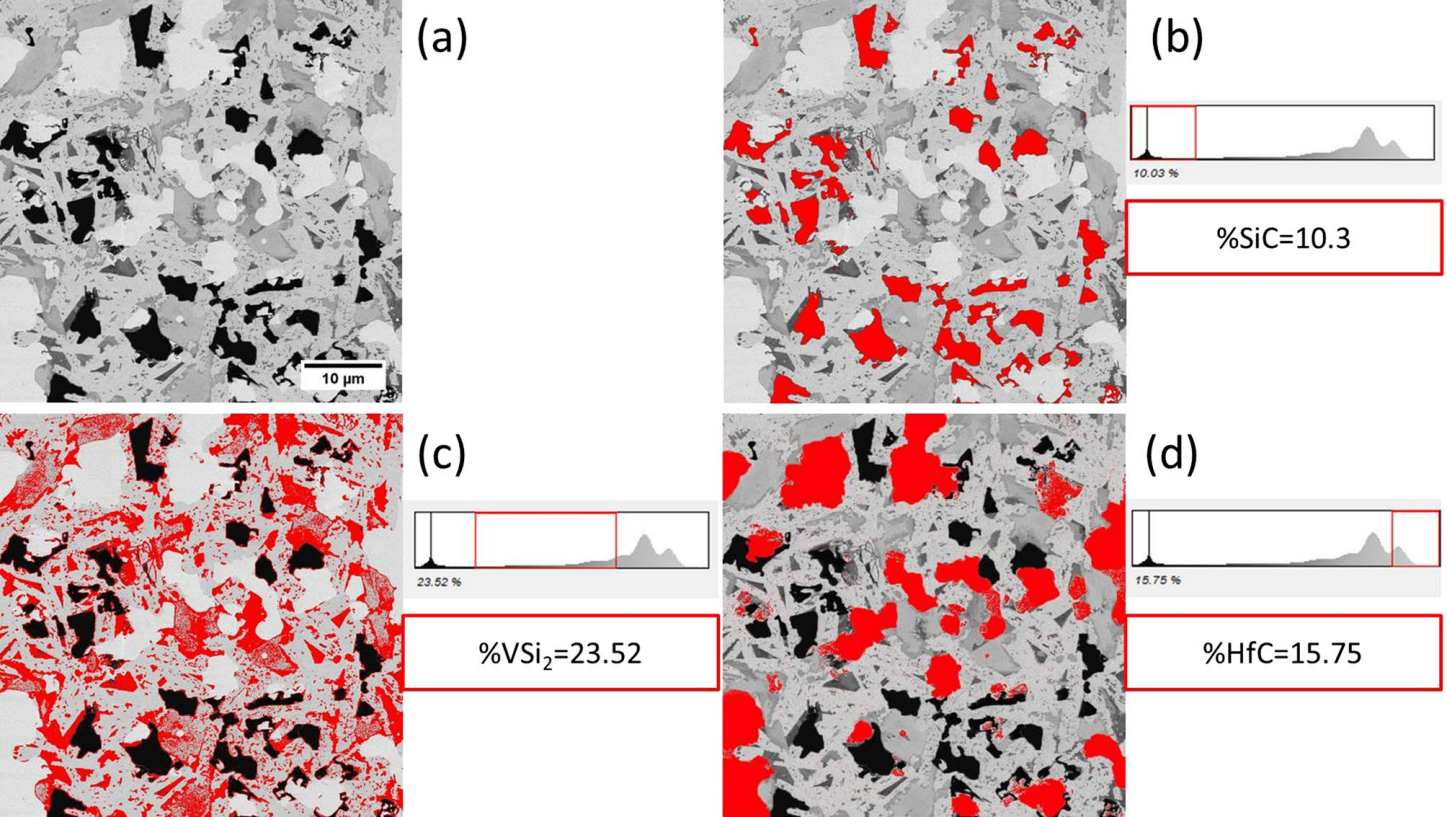
Estimated chemical composition of the pressureless sintered composite by ImageJ software. (**a**) the analyzed micrograph of HfB_2_–SiC–VSi_2_-composite, (**b**) estimated composition of SiC to 10.3%, (**c**) estimated composition of VSi_2_ to 23.52%, and (**d**) estimated composition of HfC to 15.75%.

Figure 2 shows the final composition of the composite after the sintering process. The final composition of the composite was estimated HfB_2_–10.3%SiC–20.52%VSi_2_–15.75%HfC by image analysis which is close to the target composition.

### Mechanical properties

The values of the Vickers hardness, the elastic modulus, and the fracture toughness of the composite are listed in Table 2. Owning to the hardness value of SiC (~ 27 GPa)^36^ and HfC (~ 28 GPa)^37^, the average hardness of the reactive pressureless sintered composite reached 20.1 GPa. However, the hardness value of VSi_2_ is lower than HfB_2_ matrix; in situ VSi_2_ phase formation improved the hardness by promoting the elimination of porosities.

Sonber et al.^38^ fabricated HfB_2_–TiSi_2_ composite by hot pressing method. They reported the hardness value of 11.5 GPa for monolithic HfB_2_ and 25.4 GPa for HfB_2_–TiSi_2_ composite.

Ghadami et al.^21^ demonstrated that the Vickers hardness of HfB_2_-based composite improves with the in situ formation of SiC and MoSi_2_ during sintering. They reported the hardness value of 18 GPa for monolithic HfB_2_ and 25.2 GPa for HfB_2_–SiC–MoSi_2_ composite. Improvement of density by the formation in situ phases as well as the inherent hardness of in situ phases contributed to the desirable Vickers hardness of the composite.

**Figure 3:**
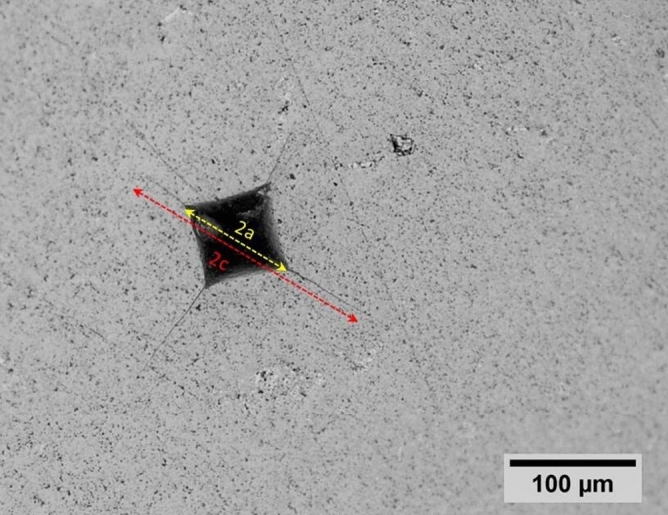
Optical microscope image of Vickers indentation for HfB_2_–SiC–VSi_2_ composite. Lengths of parameters a and b are shown based on Eq. (2).

Young’s modulus of the composite was 401.3 GPa in which was close to the estimated Young’s modulus using the role of mixture (about 424.7 GPa). According to Fig. 3, to evaluate the fracture toughness of the composite, the average half-length of the cracks was measured 45 μm and the average half-length of indentation diagonal was measured 108 μm.

**Figure 4:**
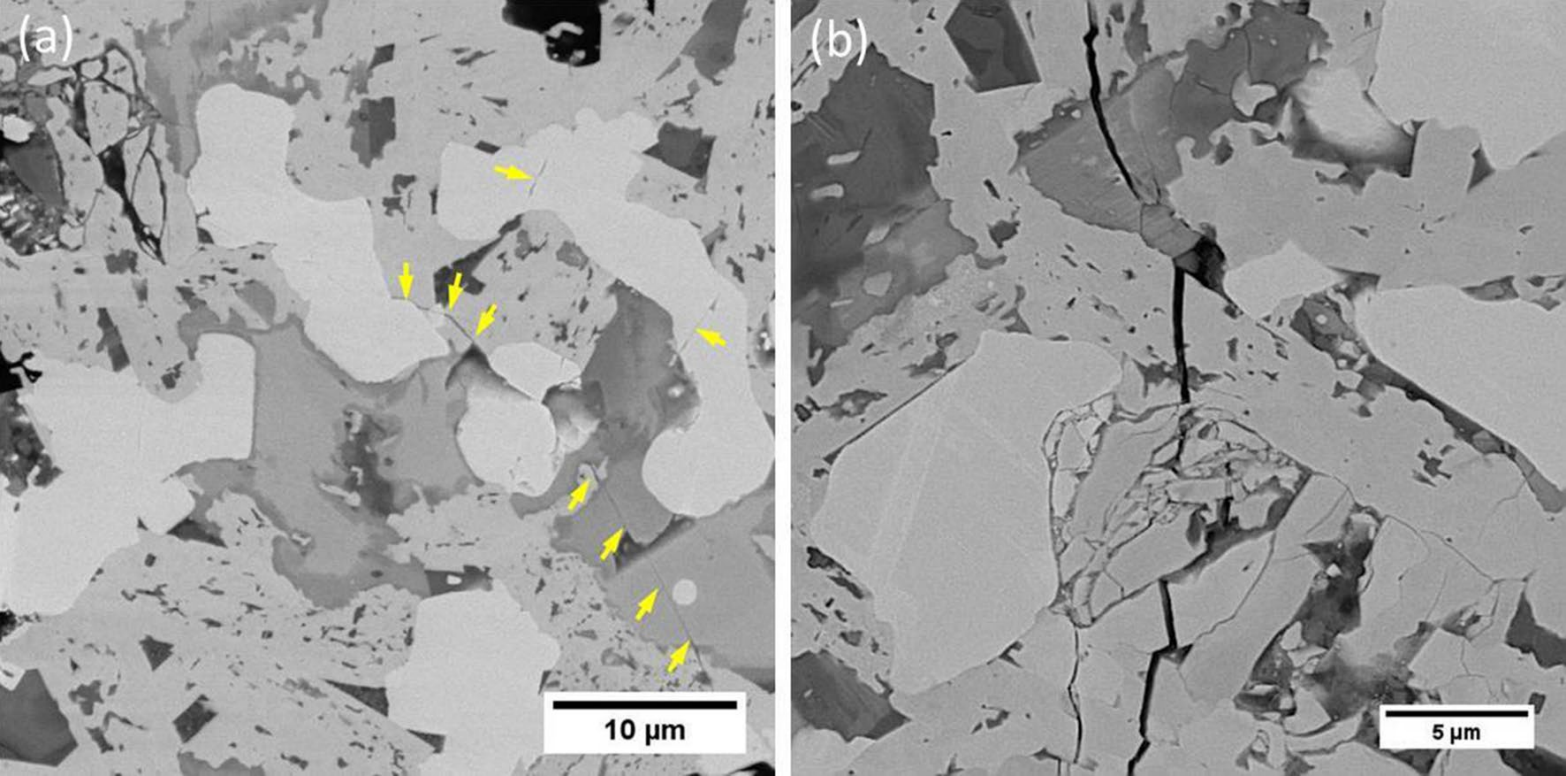
Backscattered image of indented crack propagation of HfB_2_–SiC–VSi_2_ composite. (**a**) low magnificent, the crack propagation is shown in yellow arrows and (**b**) high magnificent.

The fracture toughness of the composite was measured to be 5.8 MPa m^−1/2^ which was noticeably higher than those of the reported HfB_2_-based composites in the range of 3.5–3.9 MPa m^−1/2^^39,40^. The main reason for desirable fracture toughness was attributed to increasing obstacles for crack propagation by in situ formations of VSi_2_ and SiC. HfB_2_ large grain size (~ 10 μm) could also increase the fracture toughness. Figure 4 shows the crack propagation in the microstructure of the composite.

Because of a significant mismatch between the thermal expansion coefficient of HfB_2_ (6.3 × 10^−6^ K^−1^)^41^, SiC (4.7 × 10^−6^ K^−1^)^42^, VSi_2_ (11.2–14.65 × 10^−6^ K^−1^)^43^, and HfC (6.6 × 10^−6^ K^−1^)^44^, some compressive stresses may be induced after the sintering process. Therefore, the SiC particles are under compressive stress. On the other side, HfB_2_ matrix is under tensile stress in a tangential direction as well as compressive stress in a radial direction. The compressive stress around SiC particles causes the crack is deflected. It should be concluded that the crack is deflected when the crack strikes SiC particle. SiC particle dissipates the energy of the crack resulting in enhancing the fracture toughness of the composite.

Hence, increasing compressive stresses around SiC particles enhanced the fracture toughness of HfB_2_–SiC–VSi_2_ composite. These results were supported by other researchers^45–49^.

In previous studies, the effective role of reinforcement morphology was demonstrated^21,50^.

The SiC particles were elongated and homogeneously distributed in the HfB_2_ skeleton.

Besides, in situ formations of needle-like SiC particles provided more obstacles against the crack propagation. Padture et al.^51^ reported that the elongated SiC grains enhance the fracture resistance by crack bridging and crack deflecting.

**Figure 5:**
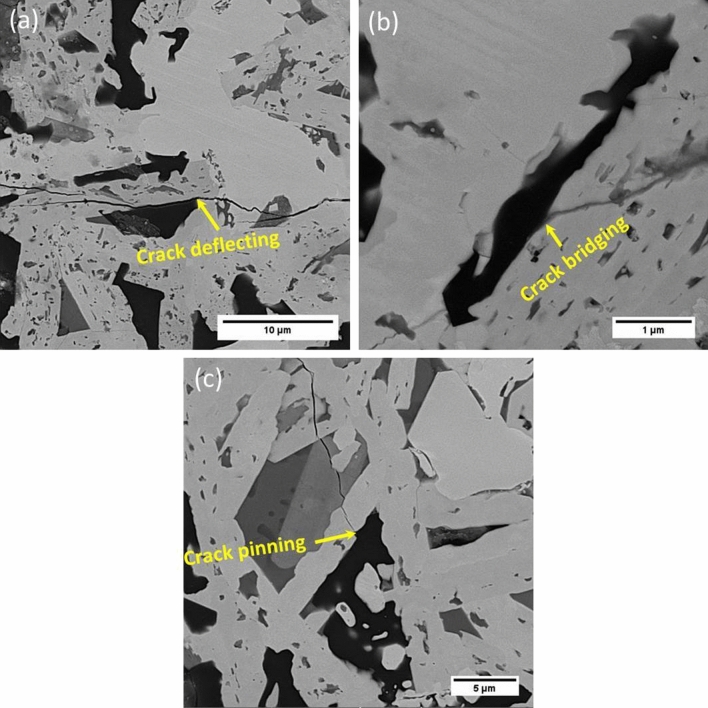
Toughening mechanism of the sintered composite. (**a**) Crack deflection, (**b**) crack bridging, and (**c**) crack pinning.

Figure 5 shows the interaction between in situ SiC particle and the crack. When the growth path of the crack tip strikes the SiC particle, three mechanisms may occur. First mechanism: the energy of the crack is not enough to break the SiC particle, but the crack has enough energy to change its growth direction. Therefore, the crack is deflected through the weaker direction (Fig. 5a). Second mechanism: in situ elongated SiC particle dissipates the crack energy by crack bridging mechanism (Fig. 5b). Third mechanism: SiC particle absorbs the whole energy of the crack and then the crack is pinned (Fig. 5c).

As a result, the formation of elongated α-SiC particles contributed to the favorable fracture toughness of the reactive pressureless sintered composite.

**Figure 6:**
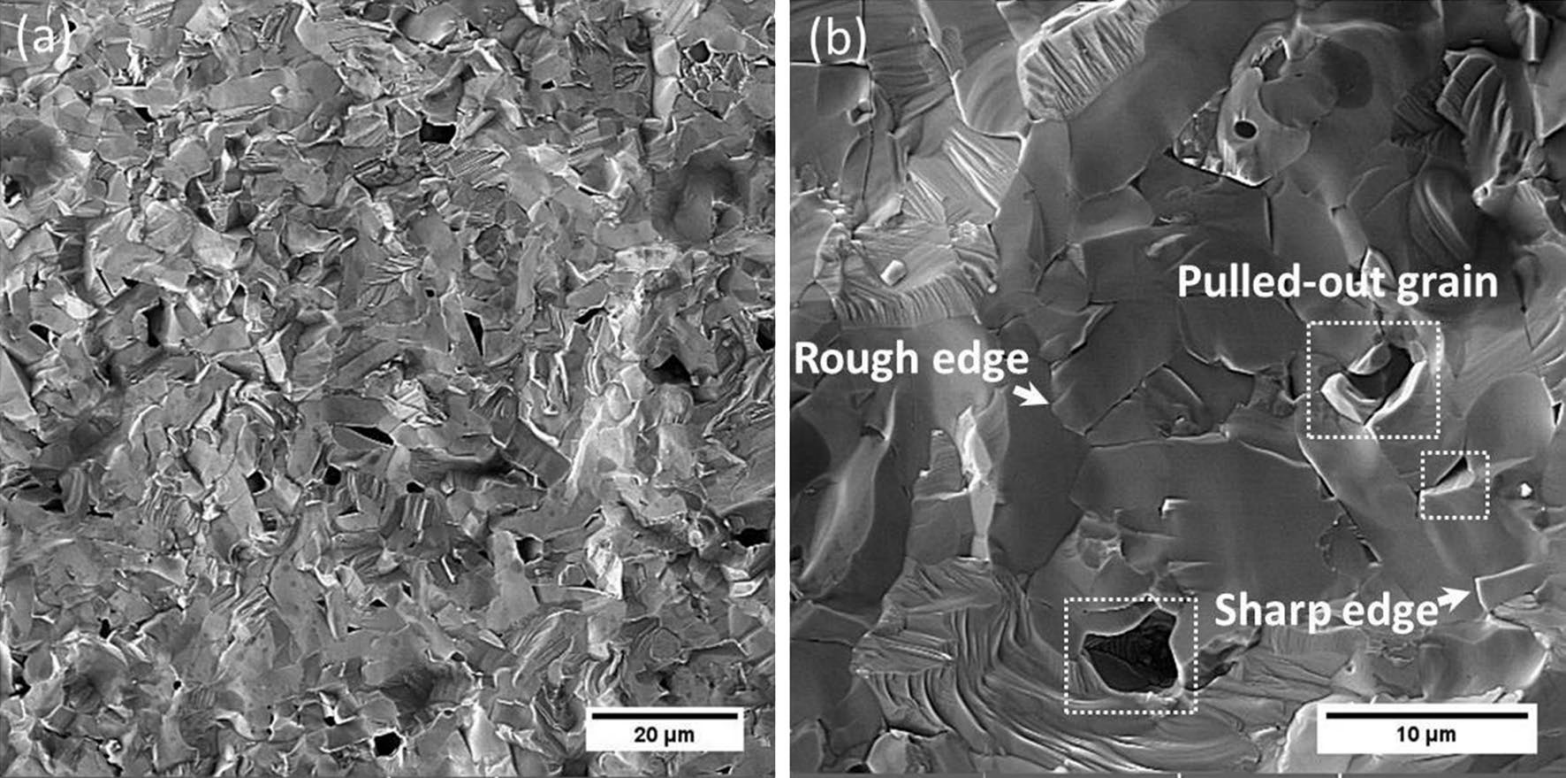
SEM fractographic of the composite. (**a**) low magnificent and (**b**) high magnificent. The presence of rough and sharp edges as well as pulled-out grains prove the mix of inter- and intra-granular fracture mode.

Figure 6 presents the fractured surface of the composite. As can be seen, in some areas the fracture surface is rough whereas in other areas the fracture surface is sharp and grains are pulled out. The sharp edges and pulled out grains prove that the crack propagates through the grain boundaries and leads to the inter-granular fracture mode. On the other side, the rough surfaces indicate that the grain boundaries are much more stronger than the inside of grains. The crack propagates through the inside of grains and leads to the intra-granular fracture mode.

This result proves that the fracture mode was mixed with inter- and intra-granular modes.

In situ formation of SiC and VSi_2_ reinforcement particles contributed to improving the strength of grain boundaries and finally enhanced the fracture toughness of the composite.

### In situ phase formation

**Figure 7:**
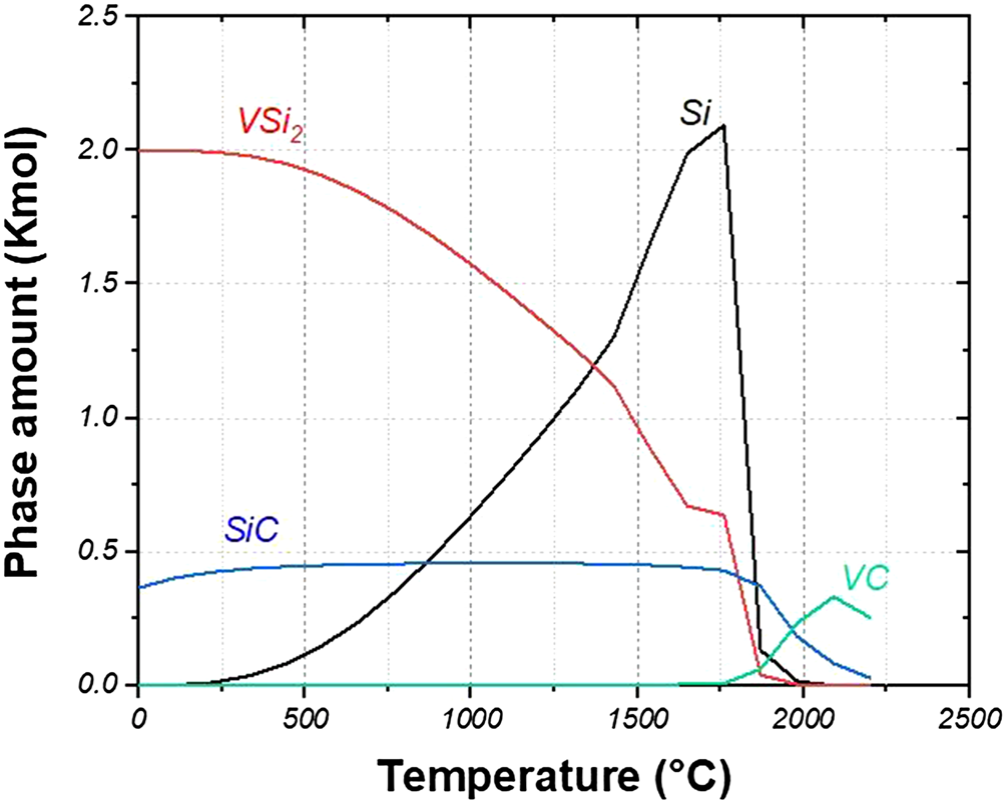
Calculated multiphase equilibrium by HSC software for in situ formation of SiC and VSi_2_ reinforcement phases according to the sintering condition (VC = 1 kmol, Si = 3 kmol, and P ~ 0.05 mbar).

Figure 7 illustrates the diagram of reaction possibility between VC and Si which was simulated according to the sintering condition (~ 0.05 mbar) by HSC software. VC and Si could react with each other and produce VSi_2_ and SiC simultaneously. According to the thermodynamic calculations, VSi_2_ and SiC could be formed even at the room temperature and the reaction (3) could happen at the beginning stages of the sintering process as following:

3 $${\text{VC}} + {\text{3Si}} = {\text{SiC}} + {\text{VSi}}_{{2}}$$

However, it seems that the required kinetic energy for activation of the reaction (3) is not adequate at the initial temperatures. Ko et al.^52^ demonstrated that SiC and VSi_2_ could be formed at 1250 °C under argon atmosphere. Hence, the formation of SiC and VSi_2_ needs higher temperatures (at least 1250 °C).

On the other side, VSi_2_ and SiC decompose at 1350 °C and 1800 °C, respectively. However, the kinetic energy of the reverse direction of the reaction (3) is not sufficiently adequate. Hence, the decomposition of VSi_2_ and SiC did not take place under the present sintering conditions (see Sect. 3.1). It leads to the conclusion that in situ VSi_2_ and SiC phases could be mostly formed at 1250 °C with ∆G of − 92.793 kJ.

Shahedi Asl et al.^53^ reported that the reaction between VC and ZrB_2_ could be possible according to the following reaction:

4 $${\text{ZrB}}_{{2}} + {\text{VC}} = {\text{VB}}_{{2}} + {\text{ZrC}}$$

Similarly, there is a chance to the reaction between HfB_2_ and VC as following:

5 $${\text{HfB}}_{{2}} + {\text{VC}} = {\text{VB}}_{{2}} + {\text{HfC}}$$

**Figure 8:**
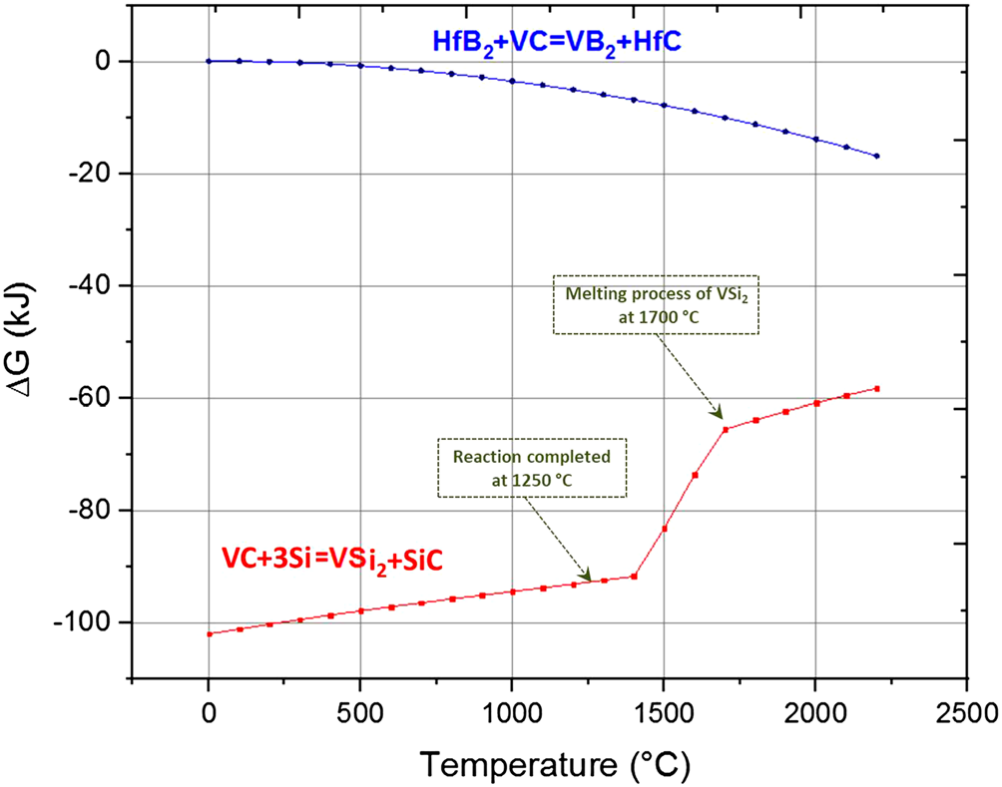
Standard Gibbs free energy of reactions between VC/Si and HfB_2_/VC as a function of temperature at standard state (P ~ 1 atm).

To find out the possibility of the reaction between HfB_2_ and VC, thermodynamic calculations were performed for the reaction (5). Figure 8 illustrates the priority between reactions (3) and (5). With a larger negative delta G for the reaction (3), the reaction (3) is progressed predominantly. Therefore, the formation of VB_2_ and HfC phases from the reaction (5) is unlikely to happen. Back to the details of the reaction (3), SiC and VSi_2_ were completely formed at 1250 °C. In the temperature range of 1400–1700 °C delta G of this reaction was dramatically increased which indicated that a thermodynamic transformation could occur. It was reported that the melting points of SiC and VSi_2_ are to be 2830 °C and 1677 °C, respectively^54,55^. It seems that the endothermic transformation is related to the melting of VSi_2_. Based on this hypothesis, the melting process of VSi_2_ was thoroughly completed at 1700 °C. This result was supported by the extracted result from the microstructural study. Moreover, in situ HfC phase could be formed according to the following reaction:

6 $${\text{HfO}}_{{2}} + {\text{3WC}} = {\text{HfC}} + {\text{3W}} + {\text{2CO }}\left( {\text{g}} \right)$$

The mass of the WC impurity from milling media was measured which indicated that ~ 5 wt%WC was incorporated into the mixed powders. It has been reported that the located HfO_2_ on the surface of HfB_2_ powders plays a barrier role against densification^21^. WC impurity from milling media could react with HfO_2_ from starting powder; hence, it could remove the oxide-impurity and finally enhance the sintering process.

### Phase analysis

**Figure 9:**
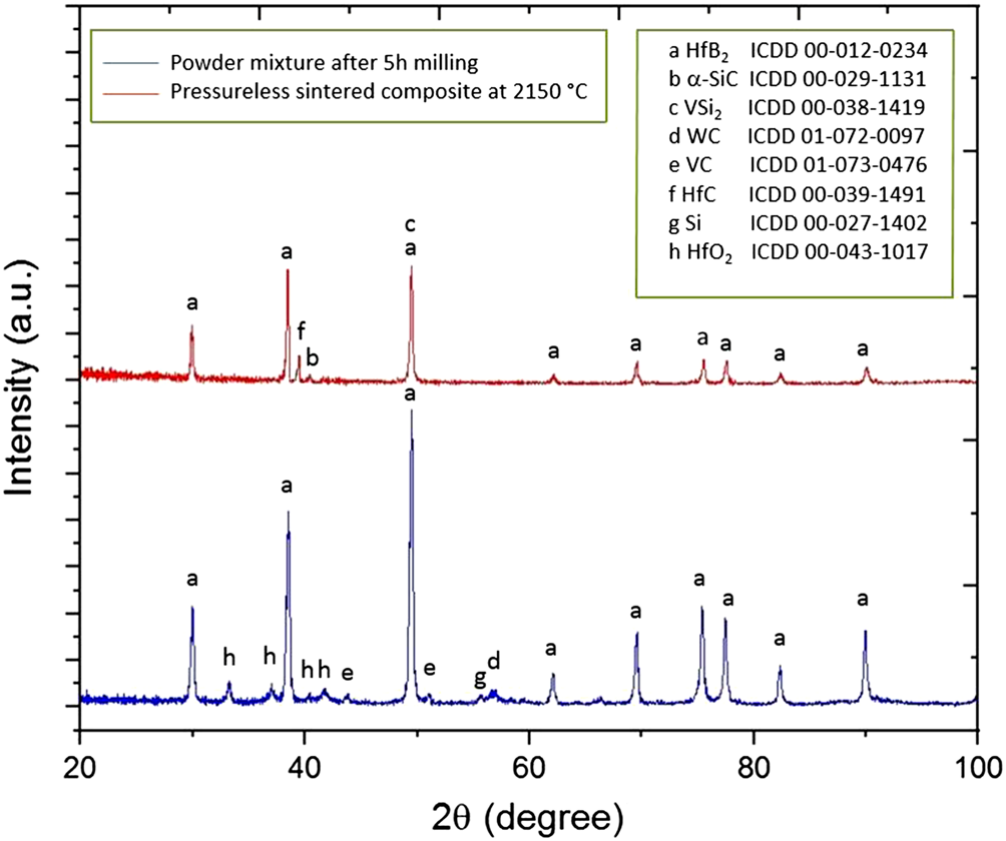
XRD patterns of mixed powders after 5 h milling process and HfB_2_–SiC–VSi_2_ composite by reactive pressureless sintering at 2150 °C.

X-ray diffraction patterns of the mixed powders and the pressureless sintered composite are shown in Fig. 9. From this Fig. 9, HfB_2_, HfO_2_, VC, Si, WC phases were detected which indicate that HfO_2_ and WC impurities were present in the starting mixtures. Owing to WC–Co cup and balls, 5 wt%WC could be inserted by the milling process. Besides, HfB_2_ powder contained HfO_2_ impurity based on Table 1. On the other hand, HfB_2_, VSi_2_, SiC, and HfC phases were found and no obvious impurity phases can be seen after the sintering process. According to the reaction (6), it could be concluded that HfO_2_ and WC reacted to each other and produced HfC. This result is supported by other researchers^56,57^.

It has been reported that carbon could be penetrated from graphite mold to the structure of HfB_2_-based composite during the sintering process^21,58^. Penetrated carbon could react with HfO_2_ impurities at the temperature of 1700 °C according to the following reaction:

7 $${\text{HfO}}_{{2}} + {\text{3C}} = {\text{HfC}} + {\text{2CO}}\left( {\text{g}} \right)$$

It should be noted that the reaction (7) as well as the reaction (6) could produce HfC. However, this work intended to fabricate HfB_2_-15vol% SiC-15 vol%VSi_2_ composite, based on the reactions (6 and 7), HfC could be formed and the final composition included HfC according to Fig. 2.

Moreover, no corresponding peaks of vanadium carbide and silicon were identified after the sintering process.

The presence of VSi_2_ and SiC peaks in Fig. 9 demonstrated that the reaction (3) was mostly completed. VB_2_ phase was not detected after the sintering process which indicated that the reaction (5) was not favorable. On the other side, HfC phase was possibly formed from the reactions (6 and 7) and less likely from the reaction (5). W phase from the reaction (6) was not detected by XRD analysis.

**Figure 10:**
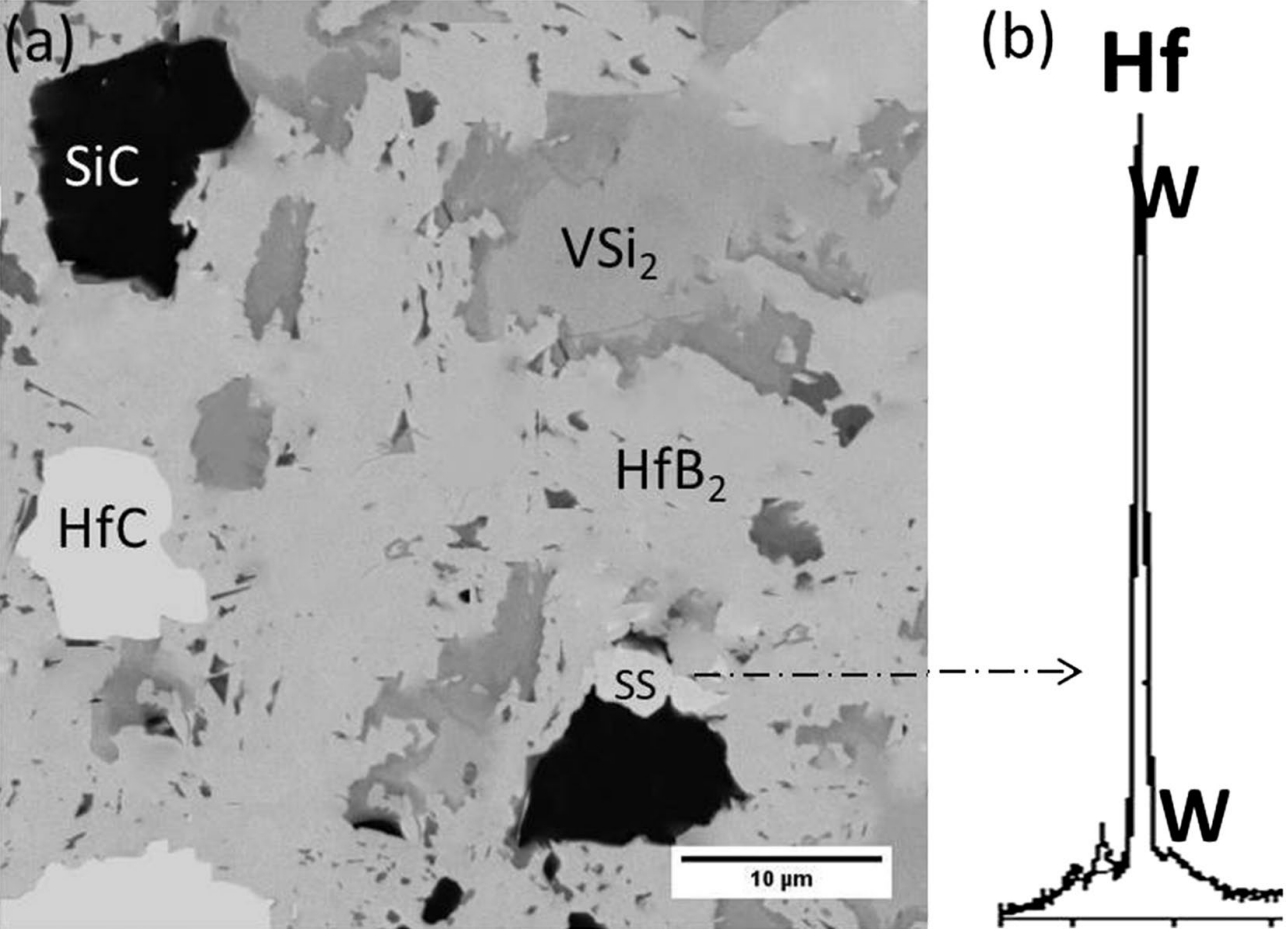
(**a**) Back-scattered electron image of HfB_2_–SiC–VSi_2_-composite and (**b**) corresponding EDS analysis showing the formation of (Hf, W)-B solid solution.

It seems that W atoms were hosted in HfB_2_ structure and led to the formation of (Hf, W)-B solid solution. However, detecting the negligible (Hf, W)-B solid solution needs to have more precise microstructural studies such as TEM technique. In this work, (Hf, W)-B solid solution was identified by EDS analysis as shown in Fig. 10. This hypothesis shows an excellent agreement with the reported result from other researchers^56,59–63^.

**Figure 11:**
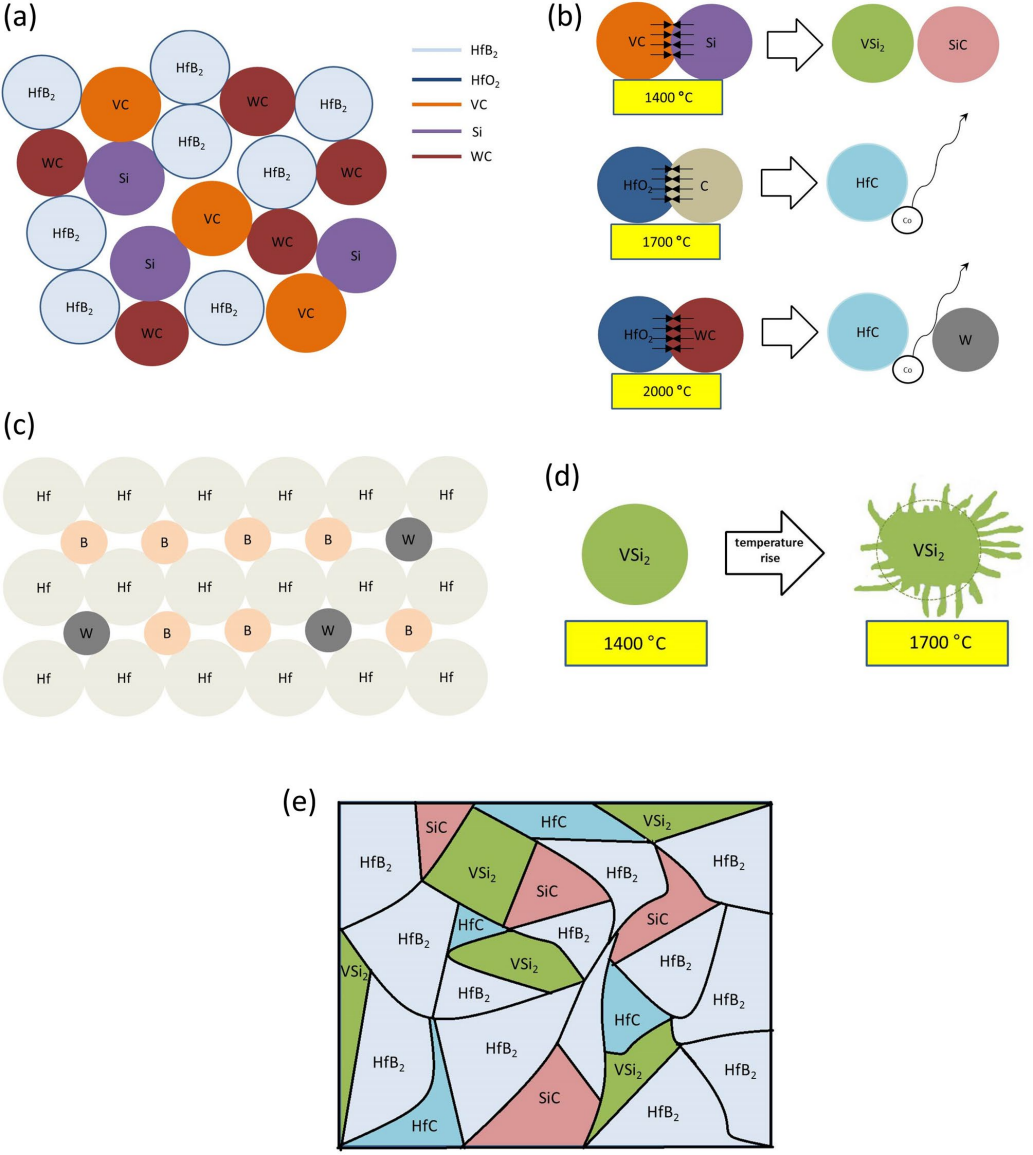
Schematic drawing of sintering mechanism during reactive consolidation of HfB_2_–SiC–VSi_2_ composite (**a**) random distribution of particles after 5 h milling process (**b**) reactions taking place during densification process (**c**) inter-substituting of W in HfB_2_ structure and formation of (Hf, W)-B solid solution (**d**) melting process of VSi_2_ at 1700 °C (**e**) final microstructure of the HfB_2_–SiC–VSi_2_ composite after sintering at 2150 °C.

Finally, Fig. 11 schematically illustrates the sintering mechanism of HfB_2_–SiC–VSi_2_ composite during the sintering process up to 2150 °C. After the milling process, mixed powders including HfB_2_, Si, and VC are randomly distributed (Fig. 11a). During the sintering process, the reaction between Si and VC could happen at 1250 °C. Therefore, VSi_2_ and SiC are formed as byproducts from reaction (3). Similarly, HfC, W, and CO(g) are formed from reaction (6 and 7) (Fig. 11b). CO gas product can release from the skeleton when HfC remains in the microstructure of the composite. W atoms from reaction (6) are hosted in HfB_2_ structure and cause to the formation of (Hf, W)-B solid solution (Fig. 11c). VSi_2_ is melted at 1700 °C and then molten VSi_2_ flows through the capillaries and fills pores (Fig. 11d). Eventually, the microstructure consists of HfB_2_, VSi_2_, SiC, and HfC phases which distribute in the microstructure after the sintering process at 2150 °C (Fig. 11e).

## Conclusions

HfB_2_–SiC–VSi_2_ composite was densified by reactive pressureless sintering using HfB_2_, VC, and Si as starting powders at 2150 °C under vacuum atmosphere (0.05 mbar) for 4 h. Microstructural investigations and XRD analysis showed that in situ SiC and VSi_2_ phases were formed during the sintering process and homogenously distributed in HfB_2_ skeleton. Moreover, HfO_2_ impurity was successfully removed and turned to HfC by reacting with inserted WC impurity from milling media. The relative density of the composite was measured by 98%. According to thermodynamic calculations performed by HSC software, VSi_2_ was melted at 1700 °C and filled pores which contributed to an increase in the relative density of the composite. The Young's modulus, Vickers hardness, and fracture toughness values of the composite were determined to be 401.3 GPa, 20.1 GPa, and 5.8 MPa m^−1/2^, respectively. The improvement of the mechanical properties of the sintered composite was attributed to the in situ formation of SiC and VSi_2_ phases.
